# Charting the Pathways of Cardiometabolic Multimorbidity: A Systematic Review of Clinical Trajectories

**DOI:** 10.3390/jcm14082615

**Published:** 2025-04-11

**Authors:** Ignatios Ioakeim-Skoufa, Rubén Ledesma-Calvo, Aida Moreno-Juste, Fátima Roque, Kerry Atkins, Miguel Ángel Hernández-Rodríguez, Mercedes Aza-Pascual-Salcedo, Francisca González-Rubio, Carmen Lasala-Aza, Óscar Esteban-Jiménez, Ana Avedillo-Salas, Celeste Cebollada-Herrera, Antonio Gimeno-Miguel, Jorge Vicente-Romero

**Affiliations:** 1 Department of Drug Statistics, Division of Health Data and Digitalisation, Norwegian Institute of Public Health, 0213 Oslo, Norway; 2 Department of Pharmacology, Physiology, and Legal and Forensic Medicine, Faculty of Medicine, University of Zaragoza, 50009 Zaragoza, Spain; 3 EpiChron Research Group on Chronic Diseases, Aragon Health Sciences Institute (IACS), Aragon Health Research Institute (IIS Aragón), Miguel Servet University Hospital, 50009 Zaragoza, Spain; 4 Drug Utilisation Work Group, Spanish Society of Family and Community Medicine (semFYC), 08009 Barcelona, Spain; 5 Research Network on Chronicity, Primary Care, and Health Promotion (RICAPPS), Institute of Health Carlos III (ISCIII), 28029 Madrid, Spain; 6 Aragon Health Service (SALUD), 50017 Zaragoza, Spain; 7 Portuguese Society of Health Care Pharmacists (SPFCS), 3000-316 Coimbra, Portugal; 8 Research Unit for Inland Development, Polytechnic of Guarda (UDI-IPG), 6300-559 Guarda, Portugal; 9 Health Sciences Research Centre, University of Beira Interior (CICS-UBI), 6201-506 Covilhã, Portugal; 10 Drug Utilisation Section, Technology Assessment and Access Division, Australian Government Department of Health and Aged Care, Canberra, ACT 2606, Australia; 11 Support and Planning Unit, Directorate of the Canary Islands Health Service, 38006 Santa Cruz de Tenerife, Spain; 12 Pharmacy Service, Virgen de la Victoria University Hospital, 29010 Malaga, Spain

**Keywords:** multimorbidity, cardiometabolic diseases, chronic disease, disease progression, comorbidity, longitudinal studies, cluster analysis, risk factors, quality of life, mortality

## Abstract

**Background:** Managing multimorbidity is a major challenge for healthcare systems. Cardiometabolic multimorbidity (CMM) is highly prevalent and linked to increased disease burden, functional decline, and mortality. While most studies focus on cross-sectional analyses, longitudinal approaches are essential for understanding disease progression and identifying patient groups who may benefit from targeted interventions. **Objectives:** This systematic review synthesises evidence from longitudinal studies on the incidence and progression of CMM, exploring transitions between multimorbidity clusters and their clinical implications. **Methods:** A systematic search was conducted in MEDLINE and EMBASE following PRISMA guidelines. Studies were included if they employed longitudinal designs and clustering techniques to assess multimorbidity evolution. The quality of evidence was evaluated using the GRADE system. **Results:** Ten studies met the inclusion criteria. CMM occurs across all age groups and both sexes, showing the highest mortality and functional decline rates. Patients with CMM frequently develop additional cardiometabolic conditions or transition to related clusters. Many also experience neurodegenerative and mental health disorders. Individuals from respiratory multimorbidity clusters often transition to CMM. Moreover, CMM is more prevalent in lower socioeconomic populations. **Conclusions:** Understanding multimorbidity trajectories enables targeted preventive strategies. Identifying patients with predictable progression can help design adequate and effective interventions, reduce health disparities, and improve healthcare outcomes.

## 1. Introduction

The concept of multimorbidity was first introduced in Germany in 1976 [1] and subsequently defined by the World Health Organisation (WHO) as the ‘*presence of two or more chronic diseases in the same person*’ [2]. The wide breadth of this definition has resulted in considerable heterogeneity in studies, particularly in terms of the number and type of diseases included [3]. Additionally, multimorbidity is often associated with other concepts such as ‘comorbidity’ and ‘frailty’. However, it should be noted that not all individuals with multimorbidity are considered frail [4]. In this context, a broader term has emerged that encompasses both multimorbidity and frailty—the ‘complex chronic patient’. This concept refers to individuals with moderate to severe illnesses who require frequent medical care, with comprehensive care being essential to address the multiple conditions that affect them [5].

Multimorbidity can significantly deteriorate the quality of life and the physical, emotional, and social well-being of the patients and their family caregivers. It is often associated with greater functional impairment, difficulties in managing polypharmacy, and an increase in adverse reactions, which often hinders adherence to treatment and increases the burden of care [6].

The prevalence of multimorbidity increases with age, affecting one in ten children, half of adults, and the vast majority of older people. In 2017, a WHO report highlighted that 70% of global deaths were caused, directly or indirectly, by chronic diseases, with cardiovascular pathologies being the main culprits [7].

The study of cardiometabolic multimorbidity, in particular, has a crucial impact on both population health and healthcare systems. Cardiometabolic patterns may manifest from early adulthood and affect both sexes [8]. A systematic association between various chronic diseases has been identified, suggesting recurrent patterns of multimorbidity. These associations are explained by different pathophysiological pathways that increase the risk of developing new diseases, although these may differ widely from a clinical perspective [8].

Despite these advances, evidence on the development and evolution of chronic diseases remains limited. There is a need to identify which pathologies tend to cluster in different population profiles and to analyse their evolution over time. This knowledge would allow more specific preventive strategies to be designed, with the aim of avoiding a greater accumulation of diseases and their associated complications.

## 2. Materials and Methods

A systematic review of peer-reviewed literature was conducted in MEDLINE and EMBASE, in accordance with the guidelines set forth in the Preferred Reporting Items for Systematic Reviews and Meta-Analyses (PRISMA) statement [9]. The search strategy employed algorithms specifically designed for multimorbidity, clustering, and longitudinal follow-up, as detailed in Appendix A.

**Figure 1:**
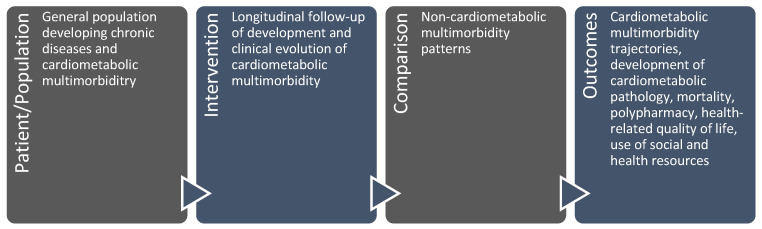
Application of the Patient/Population, Intervention, Comparison, and Outcomes (PICO) model to assess the suitability of the identified articles for inclusion in the systematic review.

In order to be included in this study, articles had to fulfil the following criteria: (i) they had to be original research articles; (ii) they had to be available in full; (iii) they had to have been published in English or Spanish; and (iv) they had to be relevant to the research question. In order to ensure that this last criterion was appropriately addressed, the Patient/Population, Intervention, Comparison, and Outcomes (PICO) framework was applied [10], as illustrated in Figure 1.

The literature review was conducted on 1 December 2024. Three researchers independently screened titles and abstracts and, when deemed necessary, the full texts, working in pairs and following a double-blind method to exclude irrelevant articles. In cases of disagreement, final decisions were reached by consensus. Additionally, the reference lists of the included studies were reviewed to identify relevant articles that met all the inclusion criteria for consideration in the systematic review.

A comprehensive range of data was collected on various study characteristics, including the year of publication, authorship, country, study period, study design, clinical setting, and study population (e.g., age and specific clinical characteristics). Additional variables included the study objectives, clustering methods for chronic pathologies, multimorbidity clusters identified at baseline and at the end of follow-up, the prevalence of these clusters, cardiometabolic trajectories (clinical course and evolution), laboratory data, drug utilisation, mortality, and polypharmacy (with definitions). Furthermore, the results of the study included the following: excessive polypharmacy (with definitions and results), inappropriate medication use (with definitions and results), health-related quality of life (with definitions and results), as well as other socio-economic and lifestyle characteristics, study limitations, and quality of evidence. The quality of evidence was evaluated using the Grading of Recommendations Assessment, Development, and Evaluation (GRADE) system [11,12].

## 3. Results

### 3.1. Literature Search Results

**Figure 2:**
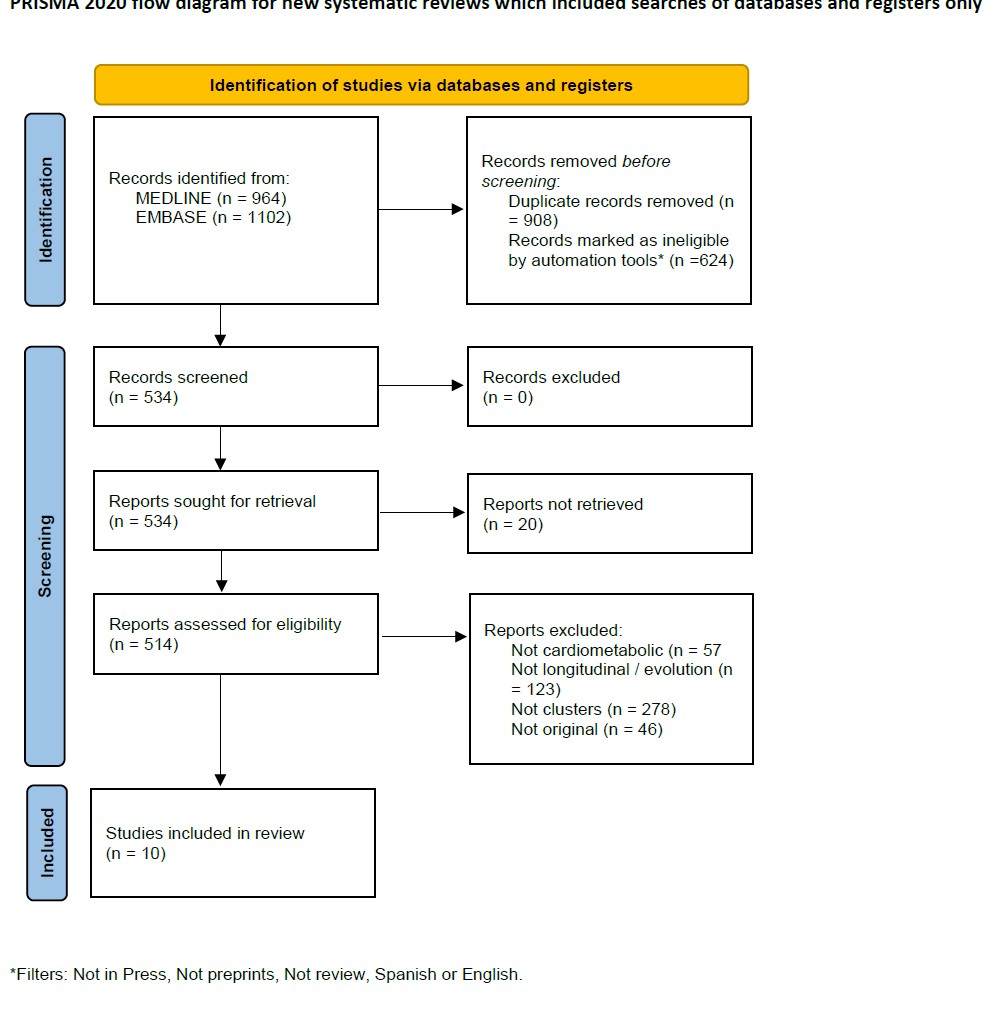
PRISMA 2020 flow diagram.

A total of 2066 studies were identified through the search conducted in MEDLINE (964 results) and EMBASE (1102 results), as illustrated in the flow chart in Figure 2. At the initial screening stage, 624 articles were excluded on the basis of automated eligibility assessment, and 908 duplicates were removed, leaving 534 publications for further review.

Of these, 20 could not be accessed. From the remaining 514 articles, 57 were excluded on the grounds that they did not address cardiometabolic issues, while 123 and 278 articles were excluded for not covering evolution and clusters, respectively. Additionally, 46 articles were excluded for not constituting original research. Ultimately, ten studies met all the inclusion criteria and were included in the systematic review.

### 3.2. Characteristics of the Studies Included

A total of ten articles were included in the systematic review (Table 1; additional information is available in Supplementary Table S1). The geographical distribution of the studies was as follows: nine were conducted in Europe, four of them in Spain, and one in the United States. All studies were observational, which limits the quality of the evidence. However, it is important to note that all studies were large-scale, population-based research projects. Seven were conducted in primary care settings, two in hospital settings, and one utilized information from both settings.

The majority of studies focus on older patients. Six studies are based on information recorded in electronic medical records; three on structured interviews, physical examinations and medical records (for clinical, functional, social, and psychological assessment), and one study is based on administrative records (e.g., billing).

The definition of multimorbidity as the presence of two or more chronic diseases is consistent across all studies. A diversity of chronic diseases is observed across the included studies, with the number of clinical entities ranging from 3 to 153. Furthermore, the number of cardiometabolic diseases also varies widely, with between 2 and 20 cardiovascular-metabolic diseases described in the identified patterns. However, studies focusing on older patients (≥60 years) tend to utilise the SNAC-K list comprising 60 chronic diseases [23].

Three primary methodologies have been identified for addressing the study of the evolution of multimorbidity: cluster analysis, Markov models, and sequence analysis (which refers to the chronological order of disease occurrence). There are also methodological differences in the criteria used to define the total number of clusters, the clinical profile of each cluster, and its descriptor (name). In most studies, a so-called ‘non-specific’ cluster was identified with a high prevalence, which may exceed 70%. The prevalence of cardiometabolic clusters in the baseline phase ranges from 7% to 63%, and in the study with the lowest prevalences of cardiometabolic clusters, prevalences of non-specific clusters were observed up to 84%.

### 3.3. Key Information About the Included Studies Using Cluster Analysis

Vetrano D.L. et al. (2020) studied a cohort of people ≥ 60 years in Sweden, with follow-ups at six and twelve years after baseline [13]. Most patients with cardiometabolic multimorbidity developed other types of cardiovascular disease, so they were classified into a similar cluster or, if not, into a different cardiometabolic pattern in subsequent controls. However, a significant percentage of them developed neuropsychiatric pathology. At the first follow-up, 15.8% of patients in the *Respiratory and Musculoskeletal diseases* cluster, as well as 14.5% of patients in the *Eye diseases and cancer* cluster, progressed to the *Cardiometabolic* cluster. At the 12-year follow-up, 20.6% of those in the *Unspecific* cluster, 19.0% of those in the *Musculoskeletal, respiratory and immune diseases* cluster, and 15.9% of patients in the *Eye diseases* cluster developed significant vascular diseases. A limitation of the study was that the diseases were considered independently of their severity.

Haug N. et al. (2020) studied 250,498 healthy individuals who developed diabetes mellitus during the follow-up period [15]. Through cluster analysis, the researchers divided the participants into ten macro-groups (clusters) and observed an evolutionary trend. They described the sequence of the onset of multimorbidity in patients with diabetes mellitus. Initially, these patients presented with a wide range of diseases, followed by the development of metabolic and eye diseases. Heart disease subsequently emerged, later combining with respiratory, cerebrovascular and renal disease. Notably, the progression of multimorbidity was faster in the group of patients with diabetes mellitus than in the control group. In addition, women progressed to high multimorbidity more quickly than men. The utilisation of medical claims data is subject to a number of limitations with regard to the study.

The study by Cezard G. et al. (2022) examined the incidence of diabetes mellitus, cardiovascular disease, and cancer in patients aged 40 to 74 years [16]. One of the main objectives of this study was to define the order of occurrence of these pathologies. During the study, 16% of participants developed diabetes, and 19% developed cancer. In addition, among those first diagnosed with diabetes, 7% later developed cardiovascular disease, while 3% developed cancer. Using cluster analysis, the researchers distinguished seven typical multimorbidity trajectories. Older age and lower socio-economic status were associated with a faster development of multimorbidity. The cluster labelled as *Fast Multimorbidity and death* comprised older patients with lower socio-economic status, who also experienced more hospital admissions. It is acknowledged that a limitation of this methodology is that, in order to identify typical trajectories using a sequence analysis approach, a number of choices must be made at the optimal matching and cluster analysis stages.

The study conducted by Carrasco-Ribelles L.A. et al. (2023) involved a dynamic longitudinal cohort derived from the electronic medical records of 1,456,052 patients aged 65 years and older [18]. Using cluster analysis, the researchers identified eleven patterns of multimorbidity, of which five were related to cardiometabolic conditions. A large proportion of patients died during the study. The cluster with the highest mortality rate was *Heart and circulatory*, with a mortality rate of 61.33%, closely followed by the *Chronic ulcers and peripheral vascular* at 56.45%. These high mortality rates in the cardiometabolic clusters over the 9-year study period may have underestimated the significance of the trends observed. At the beginning of the study, most patients in the cardiometabolic clusters remained in the same cluster or transitioned to another cardiometabolic cluster after nine years. However, a notable percentage of them shifted to the *Mental and neurodegenerative* and *Neuromusculoskeletal* clusters. Additionally, the multimorbidity pattern with the highest likelihood of developing cardiometabolic diseases was the *Genitourinary and respiratory* cluster. On the other hand, the *Cardiometabolic disease* pattern had the highest rates of severe frailty. Along with the *Chronic ulcers and peripheral vascular* and *Mental and neurodegenerative* cluster, it also had a greater risk of requiring home care. It is important to note that certain chronic conditions or frailty deficits may be under-reported among patients who visit primary care centres less frequently. This phenomenon constitutes an information bias.

In the EpiChron cohort, which includes all users of the Aragon National Health System, Ioakeim-Skoufa et al. (2024) investigated the evolution of 293,923 young and middle-aged Spanish patients aged 18 to 65 years with multimorbidity [21]. They employed cluster analysis at baseline, as well as at four and eight years of follow-up. At baseline, the researchers identified and clinically described three patterns of multimorbidity, but two additional patterns emerged in subsequent controls. The identified cardiometabolic clusters were *Dyslipidaemia and endocrine-metabolic* and *Hypertension and obesity.* Notably, two out of three chronic patients with multiple conditions exhibited a cardiovascular clinical profile during the follow-up. The most common transitions observed were among cardiometabolic clusters, while a smaller percentage of patients developed anxiety and depressive disorders. Importantly, the *Hypertension and obesity* cluster had the highest mortality risk, with a death rate of 5.54% over the 8-year study period. The study lacked information on various factors that can influence health outcomes, such as biochemical data (lab tests) and various demographic and socio-economic characteristics of particular interest.

In the study by Lleal M. et al. (2024), a cohort of 3988 patients aged > 65 years, with suspected and confirmed COVID-19, was included [22]. Six clusters of chronic multimorbidity were identified ten years prior to baseline, while five clusters were identified both five years prior to the baseline and at baseline. One of the groups that showed high consistency across all time points was the *Metabolic and vascular diseases*, characterised mainly by diabetes mellitus and peripheral/visceral vascular disease. This cluster had the highest proportion of men at baseline and five years earlier. The gradual accumulation of new diseases among participants indicated that most individuals in the *Male-predominant diseases* and the *Lipid metabolism disorders* clusters moved to the *Unspecific* cluster during the first transition. A number of factors were not considered in this study, including frailty, geriatric syndromes, chronic medication, and care received.

### 3.4. Key Information About the Included Studies Using Markov Models

Violán C. et al. (2020) studied the evolution of multimorbidity patterns in people aged 65–99 years in Catalonia, Spain, collecting information from primary care electronic medical records [14]. Their research identified ten multimorbidity clusters, four of which were cardiometabolic, and analysed the changes in these patterns over a five-year period. Most patients remained within the same cluster they were assigned to at baseline. Notably, clusters C4 (*Cardio-circulatory and renal*), C6 (*Nervous, digestive and circulatory*) and C5 (*Cardio-circulatory, mental, respiratory and genitourinary*) exhibited the highest mortality rates, at 37.1%, 31.8%, and 28.8%, respectively. In addition, cluster C5, which comprises a diverse range of pathologies, was most prevalent in areas with lower purchasing power. Regarding the development of cardiometabolic pathology, 42% of patients were classified in cluster C1 (*non-specific*) at baseline, indicating some challenges in initial classification. Nevertheless, 10.5% of this cluster developed cardiometabolic pathology within the five-year period. A limitation of the technique is that Markov models are more effective over longer periods of time.

Roso-Llorach A. et al. (2022) studied the development of multimorbidity in 3363 elderly patients (≥60 years) in Sweden using a 12-year cohort [20]. They categorized the participants into three groups according to age at baseline: sexagenarians (aged 60–69), septuagenarians (aged 70–79), and octogenarians (aged 80 and older). Using hidden Markov models, the researchers identified four patterns of multimorbidity. Notably, two of these clusters were cardiometabolic across all age groups. The multimorbidity pattern with the shortest duration in all age groups was the *Unspecific* cluster. In contrast, the pattern with the longest duration varied between groups: it was predominantly *Psychiatric, endocrine and sensorial* in sexagenarians, *Neuro-vascular and skin-sensorial* in septuagenarians and *Respiratory-circulatory and skin* in octogenarians. Cardiometabolic patterns showed the highest mortality rates in all age groups, which may have contributed to shorter length of stay in these clusters. Furthermore, patients with a cardiometabolic profile had increased medication use, higher instances of polypharmacy, and a slower walking speed, indicating greater frailty. Lastly, the time spent in the same cluster was found to be inversely proportional to age. A notable limitation was the stratification of the study sample into three distinct age groups, which resulted in the inclusion of a limited number of individuals in certain patterns.

### 3.5. Key Information About the Included Studies Using Other Techniques

Velek P. et al. (2022) conducted a study involving 6094 healthy Dutch participants to observe their clinical course and identify the order of occurrence of the first three diseases [17]. Among the participants, 19.5% experienced a cardiometabolic pathology as their first disease, and in 19.4% of these cases, the second disease was also a cardiometabolic pathology. A significant percentage of individuals in the *Depression, Cancer*, and *Lung disease* clusters developed cardiometabolic conditions. Patients initially diagnosed with cardiometabolic pathology primarily developed other types of cardiovascular conditions, while a notable percentage developed neurodegenerative and lung diseases. It is possible that the results have been underestimated, and this may be related to incomplete participant data.

Quiñones A.R. et al. (2023) classified patients aged 45 years and older in the baseline phase of their study into five clusters based on their clinical profile, with only one cluster identified as *Cardiometabolic* [19]. A notable observation among patients with cardiometabolic multimorbidity was the tendency to develop additional cardiometabolic conditions while remaining in the same cluster. Specifically, 14% of patients in the *Cardiometabolic* cluster progressed to the *Mental and somatic* cluster. Furthermore, patients with lower economic status had significantly higher rates of chronic disease accumulation. The utilisation of diagnostic tools for the measurement of disease accumulation may not align precisely with the development of multimorbidity in individuals or populations.

## 4. Discussion

This systematic review aims to examine the characteristics of multimorbidity patterns associated with a cardiometabolic profile. We focus on two key aspects: the development (incidence) of cardiometabolic multimorbidity and the evolution (progression) of patients with this condition.

The techniques most commonly employed in these studies include cluster analysis [13,15,16,18,21,22], Markov models [14,20], and sequence analysis [16,17]. The choice of the analytical method has an influence on the number of clusters/groups and their nature [16,24]. In multimorbidity clustering, cluster analysis is used to group similar data points based on shared characteristics. This approach helps identify hidden patterns in complex datasets. K-means assigns individuals to a single cluster based on their most dominant disease patterns. This approach is useful for identifying distinct multimorbidity profiles. However, it may oversimplify complex cases where individuals have overlapping conditions. On the other hand, fuzzy c-clustering allows for partial membership, recognising that patients can belong to multiple multimorbidity clusters at the same time. This flexibility is essential for capturing shared risk factors and the interactions between diseases [20,25]. Hidden Markov models provide a dynamic perspective by modelling the probabilistic transitions between various health states. They treat the diseases in each person as random variables that are influenced by a hidden state or cluster. This makes hidden Markov models particularly valuable for understanding disease trajectories, predicting the progression of multimorbidity, and identifying critical points for early intervention. Sequence analysis offers another useful approach by examining the chronological order of disease occurrences, which facilitates a deeper understanding of multimorbidity patterns and long-term health outcomes. This non-parametric method is commonly used in social sciences to analyse trajectories and social processes. It offers a holistic view of how these processes evolve over time and the points at which transitions occur, although it can be computationally expensive when studying many disease combinations [26,27].

### 4.1. Incidence and Progression of Cardiometabolic Multimorbidity

The prevalence of cardiometabolic patterns at baseline varies significantly across studies, ranging from 7% to 63%. This discrepancy can be attributed to various factors, including methodological differences (such as lists of chronic diseases, clinical setting, data source, statistical technique used, definition of the number of clusters and clinical description) and differences in the characteristics of the study population (demographic, geographic, temporal, environmental, socio-cultural, characteristics of the health care system, etc.). The diversity of methodologies used in the identified studies may partially explain the variations in cluster prevalences. For instance, some studies report a prevalence of a non-specific cluster exceeding 70%.

Despite these notable differences, there is consensus on several important and clinically relevant conclusions: (i) during follow-up, most patients tend to remain stable within the multimorbidity pattern assigned at baseline; (ii) patterns of cardiometabolic multimorbidity are identified across all age groups and both sexes; and (iii) although there are differences in the nature (clinical description) and prevalence of the identified clusters, cardiometabolic multimorbidity clusters are associated with increased mortality, a higher risk of additional comorbidity, and other adverse health outcomes.

Regarding the development of incident cardiometabolic pathology, there is a significant association between specific multimorbidity clusters and the subsequent emergence of cardiometabolic diseases [13,14,15,16,17,18,19,20,21,22]. For example, the cluster that showed the highest tendency to develop this type of pathology includes respiratory diseases. The link between cardiovascular and respiratory pathology is well documented in the medical literature [28,29,30,31]. However, patients in other clusters also showed a significant trend towards cardiometabolic patterns, such as the non-specific cluster (which generally has a very high prevalence), the musculoskeletal cluster, the eye cluster and the cancer cluster [13,16,18,20,22].

Patients with cardiometabolic pathology often develop other cardiac and metabolic comorbidities, remaining within the same cluster during follow-up or evolving into other clusters characterized by different cardiometabolic profiles [13,14,15,16,17,18,19,20,21,22]. This observation aligns with findings from other studies [32,33]. Lappenschaar M. et al. (2013) concluded that the likelihood of developing a new chronic cardiovascular disease increases in direct proportion to the number of cardiometabolic pathologies already present in the individual [33]. Our review suggests that a significant percentage of these patients will progress to clusters of psychiatric and neurodegenerative disorders, with anxiety-depressive disorders being the most prominent. Other studies have shown that patients with cardiometabolic multimorbidity have a higher risk of developing Parkinson’s disease [34]. Furthermore, various studies have linked diabetes mellitus and multimorbidity to an increased risk of progression to kidney [6] and liver disease [35]. All articles included in this review show that cardiometabolic clusters often exhibit high mortality rates. Other studies have also shown that groups with cardiometabolic disease had the highest mortality rates [34,36,37,38,39]. These patterns typically also show greater levels of functional impairment [14,15,18,21].

### 4.2. Additional Factors: Sex, Polypharmacy, and Socio-Economic

Regarding sex differences, several studies included in this review reveal significant differences between men and women in the development of multimorbidity. Women are more likely to develop multimorbidity and, once it occurs, tend to progress more quickly to a high multimorbidity burden [15,16,17]. However, this aspect remains somewhat unclear, as other studies report no significant differences between sexes. For instance, the study by Strauss V.Y. et al. (2014) found that being female was only slightly more associated with the development multimorbidity [40], while Lappenschaar M. et al. (2013) reported no difference between men and women [33]. Moreover, the sex differences vary by age group. In early life, women carry a higher burden of multimorbidity, but these differences diminish with age. Notably, there are no significant differences in the population aged 90 and over [30,41,42].

Polypharmacy is a common issue among individuals over the age of 60, particularly in those with cardiometabolic patterns, which are also associated with increased drug usage [20]. Additionally, there is an increase in dependency and frailty rates within this group [18].

Regarding socio-economic factors, several studies included in the review highlighted the association between cardiometabolic multimorbidity clusters and lower purchasing power. Violán C. et al. (2020) found that cluster C5 (cardiometabolic profile and high multimorbidity) was most prevalent in areas of lower wealth [14]. Furthermore, Cezard G. et al. have suggested that individuals with lower wealth and socio-educational status tend to accumulate more comorbidities, experience more hospital admissions, and face a higher risk of death [16]. The existing literature provides evidence that links lower socio-economic status with higher rates of multimorbidity. Singh-Manoux A. et al. (2018) observed that clinical risk factors, such as personal and family history of cardiovascular disease, are better predictors of the risk of developing cardiometabolic disease in populations without multimorbidity [43]. Socio-economic status and lifestyle (alcohol consumption, smoking, diet, and exercise) are stronger predictors of progression to incident disease and mortality in people with cardiometabolic disease. Similarly, Rojas-Huerta A. et al. (2022) reported that the highest rates of multimorbidity were observed in individuals lacking education, stable partnerships, employment, and experiencing poor economic conditions and quality of life [44]. Moreover, differences in healthcare systems, such as screening programs for specific chronic diseases like breast, prostate, and colorectal cancer, and variations in access to these screenings among different socio-demographic groups may help explain the differences in chronic disease prevalence based on gender, age, race, and ethnicity.

Lifestyle factors, such as physical activity, diet, and stress management, play a significant role in the risk of developing various chronic diseases, including cardiovascular and metabolic conditions. Unfortunately, this important information is not always consistently recorded in clinical histories, which limits the effectiveness of many research studies. By examining these lifestyle factors more closely, we could gain valuable insights into the onset of several chronic diseases [19].

### 4.3. Limitations Ans Strengths

This work has some limitations commonly found in systematic reviews. These include the breadth of the literature search (for which we opted for a broad search to minimise the loss of relevant studies), the databases used (we only used MEDLINE and EMBASE), language bias (we only included articles in Spanish and English), reviewer bias, and publication bias, taking into account that non-significant results are less likely to be published. An additional limitation is the considerable heterogeneity among the articles, highlighting the variability of the methods used, designs and population characteristics, which complicates the synthesis and comparison of the results. The lack of important data, such as medication use, laboratory data, socio-economic and cultural variables, and patient preferences, may also limit the ability to draw robust conclusions. Another important limitation of the study is that it only includes observational studies with a low degree of certainty of evidence, according to GRADE. Therefore, due to the low certainty of evidence, we cannot extrapolate the results, but the data observed in the different studies allow us to draw some conclusions that can serve as a guide for clinical practice and future research.

On a positive note, a strength of this study lies in its adherence to standardised and rigorous methodology according to PRISMA guidelines. We conducted a thorough literature search using well-defined terms in the two largest existing databases (MEDLINE and EMBASE). We also stablished clear inclusion and exclusion criteria that facilitated a transparent selection of studies, thereby enhancing the validity and reproducibility of the results.

### 4.4. Clinical Implications and Future Research Recommendations

It is well-known that the accumulation of chronic diseases in the same individual is not random, but that there are pathologies that tend to occur systematically at the same time. Nevertheless, current evidence on these associations is still limited and warrants further investigation. Moreover, studies using clustering techniques to identify the most common groups of diseases in the population remain scarce, and most of these studies have a cross-sectional design, which complicates the exploration of causal relationships. Finally, it is important to note that while much research on the prevalence of multimorbidity has focused on older adults, a significant percentage of younger individuals also experience multimorbidity. Notably, middle-aged individuals show considerable prevalence, especially concerning cardiometabolic conditions. Investigating multimorbidity patterns offers valuable opportunities for identifying groups of patients with similar health trajectories who may benefit from the application of specific and individualised preventive interventions. Through such strategies, the aim is to reduce the accumulation of diseases in our population, lower their mortality rates, and improve the quality of life. In the context of an ageing population, preventing multimorbidity is a fundamental element of ensuring the sustainability of social and health systems.

While comparing multimorbidity patterns across studies can be challenging due to variations in methods, data sources, population structures, and disease analyses, a consistent trend emerges when examining the evolutionary aspects of patients with cardiometabolic multimorbidity [14]. To enhance the reliability of future research, several specific directions should be considered. First, it is essential to develop standardised definitions of multimorbidity to ensure consistency across studies. Additionally, harmonising clustering methodologies would help produce comparable and reproducible results, facilitating a deeper understanding of common disease patterns. Finally, future studies should more effectively integrate socio-economic determinants, such as income, education, and access to healthcare, into multimorbidity research. Real-world evidence studies may be optimised according to certain models [45]. This approach will provide a more comprehensive view of the factors influencing health outcomes. By addressing these aspects, research can better uncover the underlying mechanisms driving multimorbidity and contribute to more targeted, individualised prevention strategies.

## 5. Conclusions

This study has identified the most commonly used techniques for longitudinal research on multimorbidity, which include cluster analysis, Markov models, and sequence analysis. In the adult population, patterns of cardiometabolic multimorbidity are observed across all age groups and in both sexes, and these patterns are associated with the highest mortality rates. Patients with cardiometabolic multimorbidity tend to develop additional cardiac and metabolic conditions. These comorbidities usually remain within the same cluster or transition into other clusters with similar cardiometabolic profiles. Additionally, a significant number of these patients also develop mental and neurodegenerative diseases, with anxiety and depressive disorders being the most prevalent. Moreover, it has been noted that individuals initially classified within respiratory disease clusters often evolve towards cardiometabolic conditions. It is important to mention that cardiometabolic multimorbidity clusters are more common in areas with lower socioeconomic status, indicating a potential connection to social inequalities. Finally, cardiometabolic multimorbidity is associated with higher drug consumption and greater functional impairment when compared to other forms of multimorbidity, underscoring its considerable clinical and social impact.

The findings of this study hold significant implications for clinical practice; however, it is critical to conduct further studies to validate these results. There is a need for more longitudinal research employing standardised methodologies, which will improve comparability across different studies. Additionally, future research should integrate a wide array of socio-economic factors, such as income, education, and access to healthcare, to paint a clearer picture of the disparities in multimorbidity prevalence and evolution. It is particularly vital to focus on younger populations, especially those grappling with cardiometabolic conditions. Moreover, a comprehensive examination of how multimorbidity shapes health trajectories is crucial, along with the development of targeted, personalised interventions to effectively tackle this escalating issue within our aging populations.

## Figures and Tables

**Table 1:** Characteristics of the included studies in the systematic review.

Author (Year)	Country	Clinical Setting	Age at Baseline	Methodological Approach	Diseases Assessed, *n* (Cardiometabolic/Total)	Additional Information Assessed	Quality of Evidence (GRADE)
Vetrano DL et al. (2020) [13]	Sweden	Community or in institutions (SNAC-K database)	≥60 years	Cluster analysis (fuzzy c-means)	16/60	Education, level of disability, walking speed, cognitive status, drug information	Low ⨁⨁◯◯
Violán C et al. (2020) [14]	Spain	Primary care (SIDIAP database)	≥65	Hidden Markov models	16/60	Socio-economic status, number of invoiced drugs and polypharmacy, visits to primary care	Low ⨁⨁◯◯
Haug N et al. (2021) [15]	Austria	Specialised care	Not defined as an exclusion criteria	Hierarchical cluster analysis	≥9/131		Low ⨁⨁◯◯
Cezard G et al. (2022) [16]	UK	Specialised care (Scottish Longitudinal Study)	40–74 years	Sequence analysis, optimal matching, hierarchical cluster analysis	2/3	Marital status, household size, education, household tenure	Low ⨁⨁◯◯
Velek P et al. (2022) [17]	Netherlands	Community-dwelling (Rotterdam Study)	≥45	Chronological sequence (first three diagnoses), combinations (pairs)	4/10	Marital status, education, smoking status, blood pressure, ancestry	Low ⨁⨁◯◯
Carrasco-Ribelles LA et al. (2023) [18]	Spain	Primary care (SIDIAP database)	65–100 years	Cluster analysis (fuzzy c-means)	16/60	Socio-economic status, visits to primary care, clinical parameters, lab tests, lifestyle (smoking status, alcohol intake), emergency admission episodes, drug information, inclusion in social assistance programs	Low ⨁⨁◯◯
Quiñones AR et al. (2023) [19]	USA	Primary care (ADVANCE database)	≥45	Clinically relevant groups	8/22	Socio-economic status	Low ⨁⨁◯◯
Roso-Llorach A et al. (2023) [20]	Sweden	Community or in institutions (SNAC-K database)	≥60 years	Hidden Markov models	16/60	Education, walking speed, cognitive status, clinical parameters, lab tests, drug information	Low ⨁⨁◯◯
Ioakeim-Skoufa I et al. (2024) [21]	Spain	Primary and specialised care (EpiChron database)	18–65 years	Cluster analysis (k-means)	≥11/153	Drug information, acute diseases	Low ⨁⨁◯◯
Lleal M. et al. (2024) [22]	Spain	Primary care (MRisk-COVID study)	65–95 years (women); 65–90 years (men)	Cluster analysis (fuzzy c-means)	≥20/73		Low ⨁⨁◯◯

## Data Availability

Data is contained within the article and Supplementary Material.

## Supplementary Materials

The following supporting information can be downloaded at: https://www.mdpi.com/article/10.3390/jcm14082615/s1, Table S1: A general overview of the studies included in the systematic review; File S1. PRISMA checklist: PRISMA 2020 main checklist and abstract checklist.

## Appendix A

Search strategy performed in EMBASE and MEDLINE.

**Table:**

**Search Algorithm in EMBASE**
‘factor analysis, statistical’/de OR ‘cluster analysis’/de OR ‘patter *’:ab,ti OR ‘cluste *’:ab,ti OR ‘networ *’:ab,ti OR ‘profil *’:ab,ti OR ‘factor analysis’:ab,ti OR ‘cluster analysis’:ab,ti OR ‘network analysis’:ab,ti OR ‘Markov’:ab,ti AND ‘evolution’:ab,ti OR ‘longitudinal’:ab,ti OR ‘progress *’:ab,ti OR ‘development’:ab,ti OR ‘trajector *’:ab,ti OR ‘transitio *’:ab,ti AND ‘multiple chronic conditions’/de OR ‘multimorbidity’/de OR ‘multiple chronic conditions’:ab,ti OR ‘multimorbi*’:ab,ti
**Search algorithm in MEDLINE**
(“factor analysis, statistical”[MeSH Terms] OR “cluster analysis”[MeSH Terms] OR “patter *”[Title/Abstract] OR “cluste *”[Title/Abstract] OR “networ*”[Title/Abstract] OR “profil *”[Title/Abstract] OR “factor analysis”[Title/Abstract] OR “cluster analysis”[Title/Abstract] OR “network analysis”[Title/Abstract] OR “Markov”[Title/Abstract]) AND (((evolution[Title/Abstract]) OR (longitudinal[Title/Abstract]) OR (progress*[Title/Abstract]) OR (development[Title/Abstract]) OR (trajectory *[Title/Abstract]) OR (transitio*[Title/Abstract])) AND (“multiple chronic conditions”[MeSH Terms] OR “multimorbidity”[MeSH Terms] OR “multiple chronic conditions”[Title/Abstract] OR “multimorbid *”[Title/Abstract]))
* Literature search performed on 1 December 2024.

## References

[1] Brandlmeier P. (1976). Multimorbidität unter den älteren Patienten in einer städtischen Allgemeinpraxis [Multimorbidity among elderly patients in an urban general practice]. ZFA (Stuttgart).

[2] World Health Organization (2008). The World Health Report 2008: Primary Health Care Now More than Ever.

[3] Prados-Torres A., Del Cura-González I., Prados-Torres J.D., Leiva-Fernández F., López-Rodríguez J.A., Calderón-Larrañaga A., Muth C. (2017). Multimorbilidad en medicina de familia y los principios Ariadne. Un enfoque centrado en la persona [Multimorbidity in general practice and the Ariadne principles. A person-centred approach]. Aten. Primaria.

[4] The Academy of Medical Sciences (2018). Multimorbidity: A Priority for Global Health Research.

[5] Gual N., Yuste Font A., Enfedaque Montes B., Blay Pueyo C., Martín Álvarez R., Inzitari M. (2017). Perfil y evolución de pacientes crónicos complejos en una unidad de subagudos [Profile and evolution of chronic complex patients in a subacute unit]. Aten. Primaria.

[6] Tambo-Lizalde E., Febrel Bordejé M., Urpí-Fernández A.M., Abad-Díez J.M. (2021). La atención sanitaria a pacientes con multimorbilidad. La percepción de los profesionales [Health care for patients with multimorbidity. The perception of professionals]. Aten. Primaria.

[7] Palladino R., Pennino F., Finbarr M., Millett C., Triassi M. (2019). Multimorbidity and Health Outcomes in Older Adults in Ten European Health Systems, 2006-15. Health Aff. (Proj. Hope).

[8] Glynn L.G., Valderas J.M., Healy P., Burke E., Newell J., Gillespie P., Murphy A.W. (2011). The prevalence of multimorbidity in primary care and its effect on health care utilization and cost. Fam. Pract..

[9] Page M.J., McKenzie J.E., Bossuyt P.M., Boutron I., Hoffmann T.C., Mulrow C.D., Shamseer L., Tetzlaff J.M., Akl E.A., Brennan S.E. (2021). The PRISMA 2020 statement: An updated guideline for reporting systematic reviews. BMJ.

[10] Miller S.A., Forrest J.L. (2001). Enhancing your practice through evidence-based decision making: PICO, learning how to ask good questions. J. Evid. Based Dent. Pract..

[11] Granholm A., Alhazzani W., Møller M.H. (2019). Use of the GRADE approach in systematic reviews and guidelines. Br. J. Anaesth..

[12] Ryan R., Hill S. How to GRADE the Quality of the Evidence. Cochrane Consumers and Communication Group. CCCG. Version 3.0 December 2016. http://cccrg.cochrane.org/author-resources.

[13] Vetrano D.L., Roso-Llorach A., Fernández S., Guisado-Clavero M., Violán C., Onder G., Fratiglioni L., Calderón-Larrañaga A., Marengoni A. (2020). Twelve-year clinical trajectories of multimorbidity in a population of older adults. Nat. Commun..

[14] Violán C., Fernández-Bertolín S., Guisado-Clavero M., Foguet-Boreu Q., Valderas J.M., Vidal Manzano J., Roso-Llorach A., Cabrera-Bean M. (2020). Five-year trajectories of multimorbidity patterns in an elderly Mediterranean population using Hidden Markov Models. Sci. Rep..

[15] Haug N., Sorger J., Gisinger T., Gyimesi M., Kautzky-Willer A., Thurner S., Klimek P. (2021). Decompression of Multimorbidity Along the Disease Trajectories of Diabetes Mellitus Patients. Front. Physiol..

[16] Cezard G., Sullivan F., Keenan K. (2022). Understanding multimorbidity trajectories in Scotland using sequence analysis. Sci. Rep..

[17] Velek P., Luik A.I., Brusselle G.G.O., Stricker B.C., Bindels P.J.E., Kavousi M., Kieboom B.C.T., Voortman T., Ruiter R., Ikram M.A. (2022). Sex-specific patterns and lifetime risk of multimorbidity in the general population: A 23-year prospective cohort study. BMC Med..

[18] Carrasco-Ribelles L.A., Cabrera-Bean M., Danés-Castells M., Zabaleta-Del-Olmo E., Roso-Llorach A., Violán C. (2023). Contribution of Frailty to Multimorbidity Patterns and Trajectories: Longitudinal Dynamic Cohort Study of Aging People. JMIR Public Health Surveill..

[19] Quiñones A.R., Hwang J., Heintzman J., Huguet N., Lucas J.A., Schmidt T.D., Marino M. (2023). Trajectories of Chronic Disease and Multimorbidity Among Middle-aged and Older Patients at Community Health Centers. JAMA Netw. Open.

[20] Roso-Llorach A., Vetrano D.L., Trevisan C., Fernández S., Guisado-Clavero M., Carrasco-Ribelles L.A., Fratiglioni L., Violán C., Calderón-Larrañaga A. (2022). 12-year evolution of multimorbidity patterns among older adults based on Hidden Markov Models. Aging.

[21] Ioakeim-Skoufa I., González-Rubio F., Aza-Pascual-Salcedo M., Laguna-Berna C., Poblador-Plou B., Vicente-Romero J., Coelho H., Santos-Mejías A., Prados-Torres A., Moreno-Juste A. (2024). Multimorbidity patterns and trajectories in young and middle-aged adults: A large-scale population-based cohort study. Front. Public Health.

[22] Lleal M., Baré M., Herranz S., Orús J., Comet R., Jordana R., Baré M. (2024). Trajectories of chronic multimorbidity patterns in older patients: MTOP study. BMC Geriatr..

[23] Calderón-Larrañaga A., Vetrano D.L., Onder G., Gimeno-Feliu L.A., Coscollar-Santaliestra C., Carfí A., Pisciotta M.S., Angleman S., Melis R.J.F., Santoni G. (2017). Assessing and Measuring Chronic Multimorbidity in the Older Population: A Proposal for Its Operationalization. J. Gerontol. Ser. A Biol. Sci. Med. Sci..

[24] Ng S.K., Tawiah R., Sawyer M., Scuffham P. (2018). Patterns of multimorbid health conditions: A systematic review of analytical methods and comparison analysis. Int. J. Epidemiol..

[25] Violán C., Foguet-Boreu Q., Roso-Llorach A., Rodriguez-Blanco T., Pons-Vigués M., Pujol-Ribera E., Valderas J.M. (2016). Patrones de multimorbilidad en adultos jóvenes en Cataluña: Un análisis de clústeres [Multimorbidity patterns in young adults in Catalonia: An analysis of clusters]. Aten. Primaria.

[26] Brzinsky-Fay C., Kohler U. (2010). New Developments in Sequence Analysis. Sociol. Methods Res..

[27] Studer M., Ritschard G. (2016). What Matters in Differences Between Life Trajectories: A Comparative Review of Sequence Dissimilarity Measures. J. R. Stat. Soc. Ser. A Stat. Soc..

[28] Carter P., Lagan J., Fortune C., Bhatt D.L., Vestbo J., Niven R., Chaudhuri N., Schelbert E.B., Potluri R., Miller C.A. (2019). Association of Cardiovascular Disease with Respiratory Disease. J. Am. Coll. Cardiol..

[29] Ioakeim-Skoufa I., Poblador-Plou B., Carmona-Pírez J., Díez-Manglano J., Navickas R., Gimeno-Feliu L.A., González-Rubio F., Jureviciene E., Dambrauskas L., Prados-Torres A. (2020). Multimorbidity Patterns in the General Population: Results from the EpiChron Cohort Study. Int. J. Environ. Res. Public Health.

[30] Ioakeim-Skoufa I., Clerencia-Sierra M., Moreno-Juste A., Elías de Molins Peña C., Poblador-Plou B., Aza-Pascual-Salcedo M., González-Rubio F., Prados-Torres A., Gimeno-Miguel A. (2022). Multimorbidity Clusters in the Oldest Old: Results from the EpiChron Cohort. Int. J. Environ. Res. Public Health.

[31] Carmona-Pírez J., Poblador-Plou B., Díez-Manglano J., Morillo-Jiménez M.J., Marín Trigo J.M., Ioakeim-Skoufa I., Gimeno-Miguel A., Prados-Torres A. (2021). Multimorbidity networks of chronic obstructive pulmonary disease and heart failure in men and women: Evidence from the EpiChron Cohort. Mech. Ageing Dev..

[32] Shi X., Nikolic G., Van Pottelbergh G., van den Akker M., Vos R., De Moor B. (2021). Development of Multimorbidity Over Time: An Analysis of Belgium Primary Care Data Using Markov Chains and Weighted Association Rule Mining. J. Gerontol. Ser. A Biol. Sci. Med. Sci..

[33] Lappenschaar M., Hommersom A., Lucas P.J., Lagro J., Visscher S., Korevaar J.C., Schellevis F.G. (2013). Multilevel temporal Bayesian networks can model longitudinal change in multimorbidity. J. Clin. Epidemiol..

[34] Ren Z., Xu Y., Sun J., Han Y., An L., Liu J. (2023). Chronic diseases and multimorbidity patterns, their recent onset, and risk of new-onset Parkinson’s disease and related functional degeneration in older adults: A prospective cohort study. EClinicalMedicine.

[35] Villén N., Guisado-Clavero M., Fernández-Bertolín S., Troncoso-Mariño A., Foguet-Boreu Q., Amado E., Pons-Vigués M., Roso-Llorach A., Violán C. (2020). Multimorbidity patterns, polypharmacy and their association with liver and kidney abnormalities in people over 65 years of age: A longitudinal study. BMC Geriatr..

[36] Han Y., Hu Y., Yu C., Sun D., Pang Y., Pei P., Yang L., Chen Y., Du H., Liu J. (2023). Duration-dependent impact of cardiometabolic diseases and multimorbidity on all-cause and cause-specific mortality: A prospective cohort study of 0.5 million participants. Cardiovasc. Diabetol..

[37] Kivimäki M., Strandberg T., Pentti J., Nyberg S.T., Frank P., Jokela M., Ervasti J., Suominen S.B., Vahtera J., Sipilä P.N. (2022). Body-mass index and risk of obesity-related complex multimorbidity: An observational multicohort study. Lancet Diabetes Endocrinol..

[38] Ho H.E., Yeh C.J., Wei J.C., Chu W.M., Lee M.C. (2022). Trends of Multimorbidity Patterns over 16 Years in Older Taiwanese People and Their Relationship to Mortality. Int. J. Environ. Res. Public Health.

[39] Sonaglioni A., Lonati C., Tescaro L., Nicolosi G.L., Proietti M., Lombardo M., Harari S. (2022). Prevalence and clinical outcome of main echocardiographic and hemodynamic heart failure phenotypes in a population of hospitalized patients 70 years old and older. Aging Clin. Exp. Res..

[40] Strauss V.Y., Jones P.W., Kadam U.T., Jordan K.P. (2014). Distinct trajectories of multimorbidity in primary care were identified using latent class growth analysis. J. Clin. Epidemiol..

[41] Evert J., Lawler E., Bogan H., Perls T. (2003). Morbidity profiles of centenarians: Survivors, delayers, and escapers. J. Gerontol. Ser. A Biol. Sci. Med. Sci..

[42] Hitt R., Young-Xu Y., Silver M., Perls T. (1999). Centenarians: The older you get, the healthier you have been. Lancet.

[43] Singh-Manoux A., Fayosse A., Sabia S., Tabak A., Shipley M., Dugravot A., Kivimäki M. (2018). Clinical, socioeconomic, and behavioural factors at age 50 years and risk of cardiometabolic multimorbidity and mortality: A cohort study. PLoS Med..

[44] Rojas-Huerta A., Giraldo-Rodríguez L., Agudelo-Botero M., Mino-León D. (2022). Differences by Sex in the Presentation of Multimorbidity: Longitudinal Study in Mexican Adults Living in the Community, 2001–2018. J. Women’s Health.

[45] Ioakeim-Skoufa I., Atkins K., Hernández-Rodríguez M.Á. (2025). Optimizing real-world evidence studies for regulatory decision-making and impact assessment in pharmacovigilance. Br. J. Clin. Pharmacol..

